# Imaging Spectrum of Congenital Spinal Abnormalities: A Pictorial Review

**DOI:** 10.7759/cureus.89036

**Published:** 2025-07-30

**Authors:** Safa Hoodeshenas, Chelsea Sparks, Tushar Chandra, Laura Hayes, Jennifer Kucera

**Affiliations:** 1 Radiology, Nemours Children's Hospital, Orlando, USA; 2 Radiology, University of South Florida, Tampa, USA

**Keywords:** caudal regression syndrome, chiari malformation, congenital abnormalities, myelomeningocele, sacrococcygeal teratoma, spina bifida, spinal cord, spinal dysraphism, spinal imaging, tethered cord

## Abstract

Congenital spine anomalies include a diverse range of structural abnormalities that, if left undiagnosed and untreated, may contribute to elevated perinatal mortality rates and significant long-term neurological complications. Their broad spectrum of imaging manifestations underscores the importance of recognizing characteristic features critical to timely and effective diagnosis and treatment. This pictorial essay reviews the key imaging findings associated with congenital spine anomalies, providing a concise and practical reference for early identification and management strategies.

## Introduction and background

Congenital spine abnormalities are associated with increased perinatal mortality and, if not properly diagnosed and treated, can lead to devastating neurological consequences. Early detection and appropriate intervention are essential for improving patient outcomes. Imaging plays a pivotal role in the evaluation of patients with suspected abnormalities and provides detailed characterization in cases of known anomalies. Spine evaluation is a routine part of the second-trimester fetal anatomy ultrasonographic survey, typically conducted between 18 and 22 weeks of gestation, and includes assessment of the vertebral column, spinal canal, and overlying skin [1]. In addition to ultrasound, fetal MRI serves as a valuable complementary modality for evaluation of the fetal spine.

This pictorial essay provides a comprehensive review of congenital spinal anomalies based on the Tortori-Donati clinicoradiological classification, highlighting key imaging features to aid in timely diagnosis and appropriate management.

## Review

Classification of spinal abnormalities

According to the Tortori-Donati clinical-radiological classification [2], spinal dysraphism is categorized into two main types based on the exposure of neural tissue: open and closed forms. Open spinal dysraphism, as seen in myelomeningocele, is characterized by exposed neural elements. In contrast, closed spinal dysraphism refers to conditions where the neural tissue is covered by skin and is further subdivided based on the presence of a subcutaneous mass. In the absence of a subcutaneous mass, the spectrum includes relatively simple anomalies such as intradural or filum terminale lipomas, as well as more complex conditions like dermal sinus tracts (DSTs), split cord malformations, and caudal regression syndrome (CRS).

Open spinal dysraphism

In open spinal dysraphisms, the neural tissue is not covered by skin, leading to CSF leakage and an increased risk of infection that demands prompt intervention. The category includes four distinct types, with myelomeningocele accounting for over 98% of cases [2].

Myelomeningoceles and myeloceles

**Figure 1:**
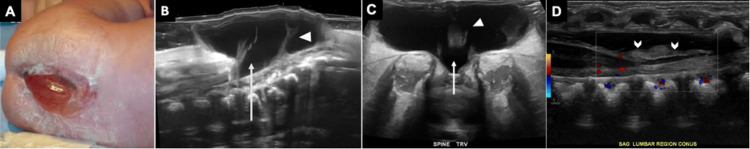
(A) Image of the clinical presentation of a myelomeningocele in an infant showing an open, cutaneous midline defect with exposure of the meninges. Sagittal (B) and transverse (C) ultrasound images of the lumbar spine with a dorsal defect and expansion of the subarachnoid space protruding through it (white arrows), containing neural tissue (white arrowheads) consistent with a myelomeningocele. (D) Additional sagittal ultrasound image more superiorly demonstrates a low-lying and stretched conus (white chevrons). All images were obtained by the authors from the institutional picture archiving and communication system (PACS). They have been de-identified and used in compliance with institutional policies.

**Figure 2:**
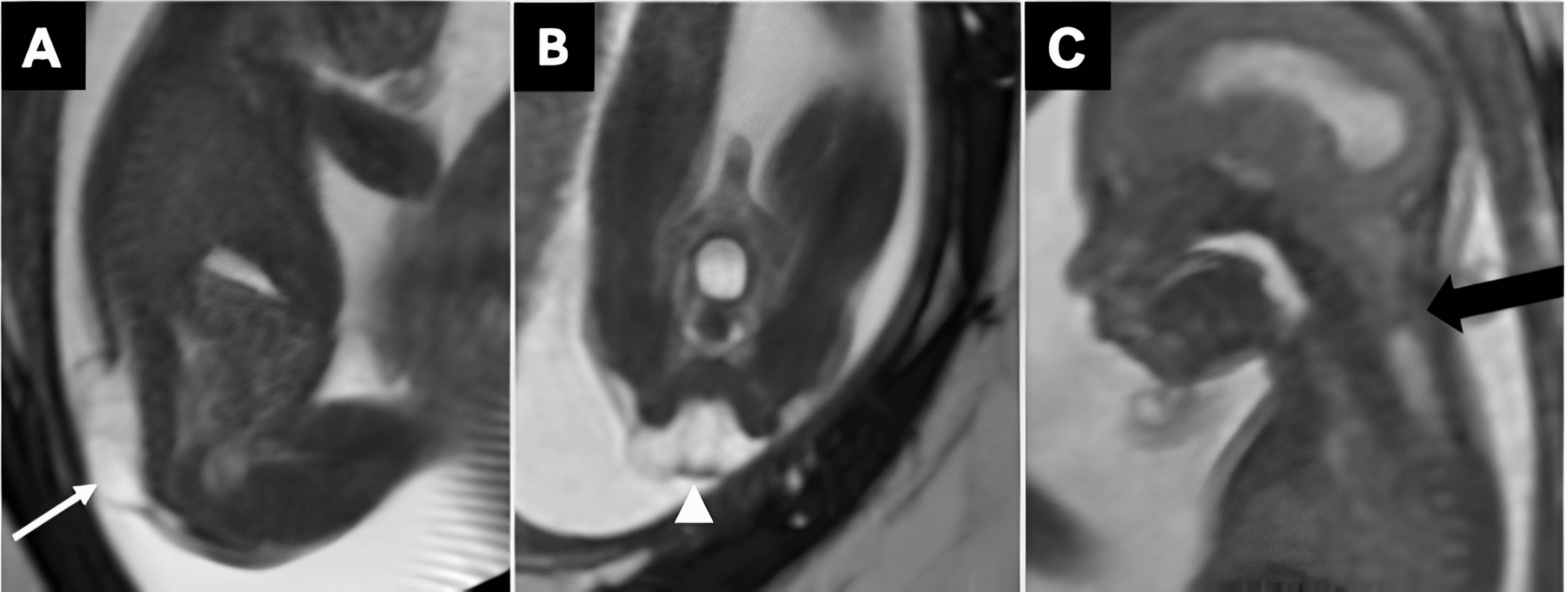
Sagittal (A) and coronal (B) T2-weighted fetal MR images of a myelomeningocele demonstrate a dorsal spinal defect not covered by skin (white arrow) and extension of the neural placode (white arrowhead) beyond the skin surface. Sagittal T2-weighted fetal MR image of the brain and upper cervical spine (C) reveals a small posterior fossa with cerebellar tonsillar herniation (black arrow), one of the features of a concomitant Chiari II malformation. All images were obtained by the authors from the institutional picture archiving and communication system (PACS). They have been de-identified and used in compliance with institutional policies.

In this subtype of dysraphism, failure of the primary neural tube to close properly results in the neural placode being exposed through a midline skin defect. In myelomeningoceles, the neural placode protrudes with meninges into a CSF-filled sac (Figure 1), while in myeloceles, the placode lies level with the skin, which makes its detection challenging during prenatal ultrasound due to the absence of a dorsal cyst in myeloceles [3]. Myelomeningoceles are nearly always associated with Chiari II malformation (Figure 2) [4].

Closed spinal dysraphism

Closed Spinal Dysraphism With a Subcutaneous Mass

**Figure 3:**
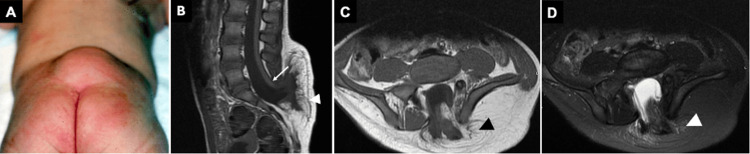
(A) Clinical presentation of lipomyelomeningocele demonstrating a subcutaneous mass above the gluteal crease. Sagittal (B) and axial (C) T1-weighted and STIR (D) MR images of a lipomyelomeningocele show a characteristic low-lying conus (white arrow) posteriorly displaced with concomitant herniation of lipomatous tissue, which is attached to the neural placode (white and black arrowheads) through the defect. STIR, short tau inversion recovery All images were obtained by the authors from the institutional picture archiving and communication system (PACS). They have been de-identified and used in compliance with institutional policies.

Lipomas with a dural defect: Lipomyeloceles and lipomyelomeningoceles are conditions caused by a defect where mesenchymal tissue enters the neural tube, leading to the development of lipomatous tissue. In these cases, the intraspinal lipoma represents a component of a larger subcutaneous lipoma that extends into the spinal canal through a broad posterior spina bifida, tethering the spinal cord. The placode-lipoma interface is situated within the spinal canal in lipomyeloceles, whereas in lipomyelomeningoceles, it is located outside the spinal canal due to the expansion of the subarachnoid space (Figure 3) [2,5].

Meningocele: A meningocele refers to the herniation of a CSF-filled sac lined by the dura and arachnoid mater. The spinal cord itself is not enclosed within the sac, though it may be tethered to the neck of the herniation [6,7].

**Figure 4:**
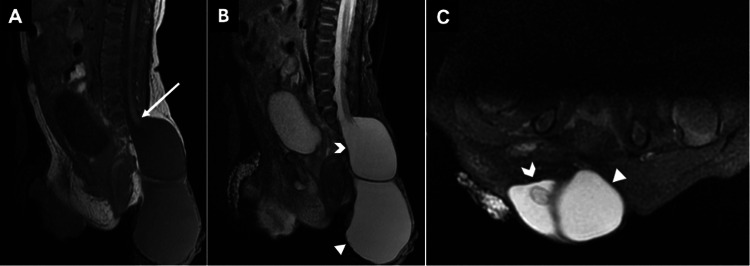
Sagittal T1 (A) and STIR (B) images of the abdomen and pelvis demonstrate a low-lying tethered conus (white arrow) with a surrounding terminal cystocele (white chevron). An additional well-defined fluid-filled structure caudal to the terminal cystocele represents a meningocele (white arrowhead), together with the terminal cystocele comprising a terminal myelocystocele. (C) Axial STIR MR image from the same patient shows the terminal cystocele (white chevron) containing the low-lying tethered conus and the adjacent myelocele (white arrowhead). STIR, short tau inversion recovery All images were obtained by the authors from the institutional picture archiving and communication system (PACS). They have been de-identified and used in compliance with institutional policies.

Terminal myelocystocele: A terminal myelocystocele is the protrusion of a large terminal syrinx into a posterior meningocele through a spinal defect. The terminal syrinx, also referred to as a syringocele, connects with the central canal but usually remains separate from the meningocele component, which communicates with the subarachnoid space (Figure 4) [6,8]. Terminal myelocystoceles can be seen in association with OEIS syndrome, which is characterized by an omphalocele, exstrophy of the cloaca, imperforate anus, and spine defects [8]. The prognosis largely depends on the presence of associated anomalies, as most children with terminal myelocystocele present with intact neurological function [2,9].

Closed spinal dysraphism without a subcutaneous mass

Simple Dysraphic States

**Figure 5:**
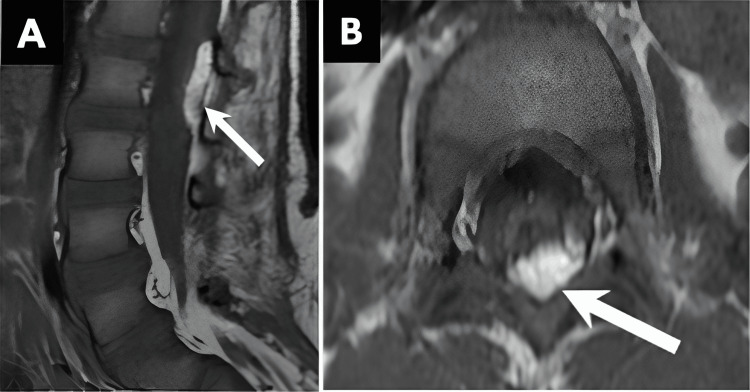
Sagittal (A) and axial (B) T1W images show a hyperintense mass compatible with an intradural lipoma along the dorsal aspect of the thecal sac at the level of L1-2. All images were obtained by the authors from the institutional picture archiving and communication system (PACS). They have been de-identified and used in compliance with institutional policies.

Intradural lipomas and filar lipomas: Intradural lipomas are most commonly found in the lumbosacral spine but can occur at any spinal level (Figure 5). Rarely, they present as intramedullary lesions or, in exceptional cases, as intramedullary lipomatosis [10,11].

**Figure 6:**
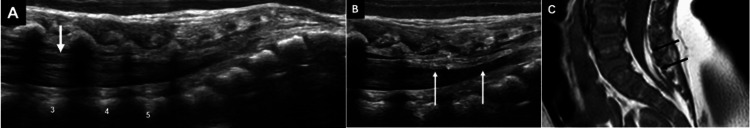
(A) Sagittal spine ultrasound shows a low-lying conus (white arrow), extending to the inferior endplate of L3, with posterior displacement. (B) A more caudal sagittal spine ultrasound image reveals an echogenic region within the filum terminale (white arrows) consistent with a filar lipoma. (C) Sagittal T1W MRI of the lumbosacral spine shows a filar lipoma, seen as a T1 hyperintense mass along the filum terminale (black arrows). All images were obtained by the authors from the institutional picture archiving and communication system (PACS). They have been de-identified and used in compliance with institutional policies.

A filar lipoma refers to fibrolipomatous thickening of the filum terminale. It is often seen in conjunction with other abnormalities, most notably type II caudal agenesis syndrome or tethered cord syndrome. A small amount of fat deposition along the filum without findings of tethered cord syndrome can be considered a normal variant (Figure 6) [6,10]. 

**Figure 7:**
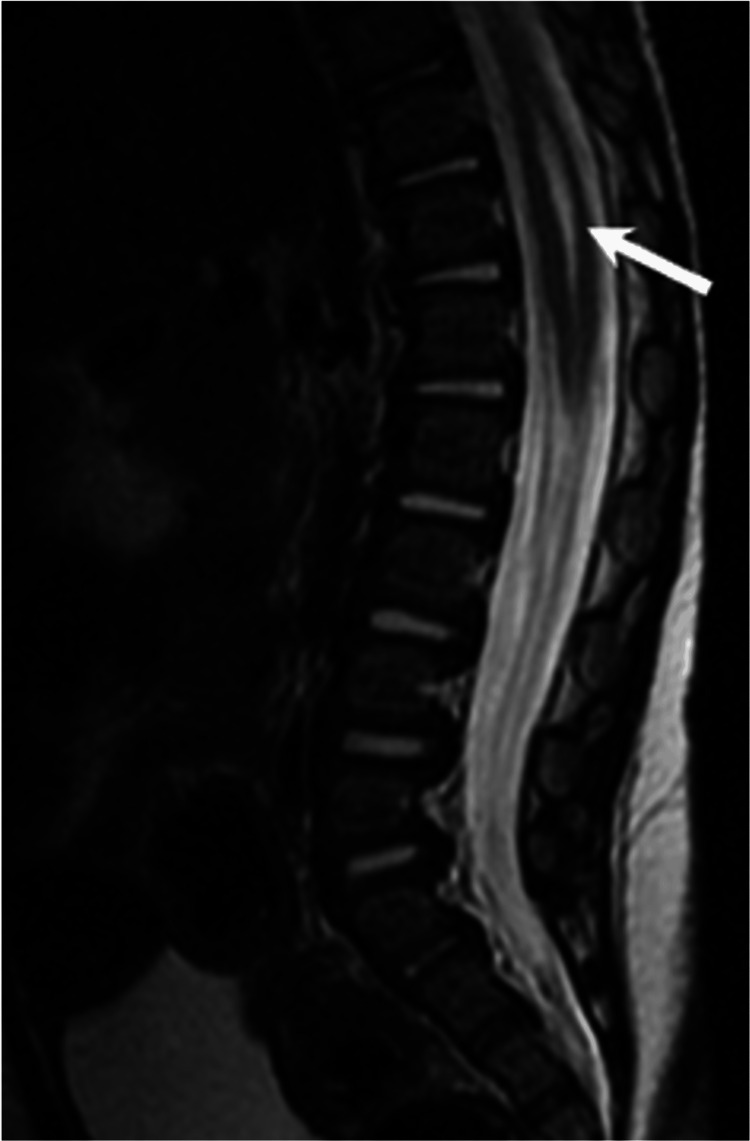
Sagittal T2W image shows a focal central canal dilatation in the region of the conus (arrow) representing a persistent terminal ventricle. All images were obtained by the authors from the institutional picture archiving and communication system (PACS). They have been de-identified and used in compliance with institutional policies.

Persistence of the terminal ventricle/ventricularis terminalis: The persistence of the terminal ventricle refers to a fluid-filled, ependyma-lined cavity located centrally within the conus medullaris, which is a persistent embryologic remnant [12]. It exhibits signal intensity similar to CSF on all sequences and does not demonstrate contrast enhancement (Figure 7). In most cases, the size of this cystic cavity remains stable on follow-up, although it may rarely enlarge [2].

Complex Dysraphic States

**Figure 8:**
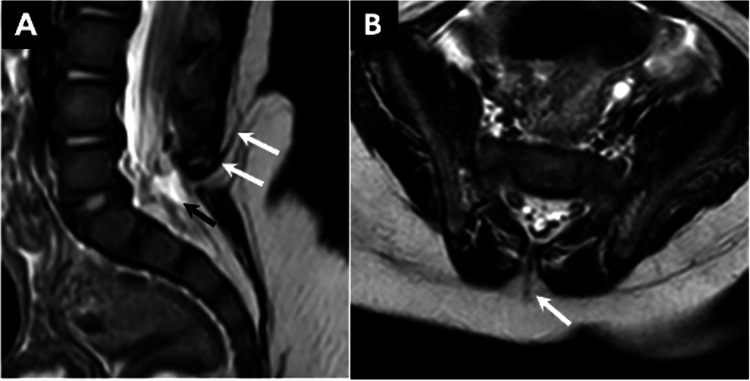
DST. Coronal T2W image (A) demonstrates a curvilinear hypointense tract (arrows) extending from the subcutaneous tissue into the spinal canal at the L5-S1 level, consistent with a DST. An 8 mm concomitant intradural lipoma at the L5 level (black arrow) and tethering of the lower cord are noted. The axial T2-weighted image (B) reveals a posterior midline osseous defect at the L5 level, with a tract connecting the posterior subcutaneous fat to the epidural fat. (white arrow). DST, dermal sinus tract All images were obtained by the authors from the institutional picture archiving and communication system (PACS). They have been de-identified and used in compliance with institutional policies.

DST: A DST is an epithelial-lined fistulous connection between the dermis and the neural tissue or meninges. As these sinus tracts connect the spinal canal to the integument, they can become a conduit for infection, possibly leading to meningitis or intraspinal abscesses (Figure 8).

DSTs can occur anywhere from the occiput to the sacrum, with the lumbosacral region being the most frequently involved and the cervical region the least commonly affected [13]. DSTs are frequently associated with other anomalies such as tethered cord, inclusion tumors, and split cord malformations [13,14]. Clinically, they can be identified as a small midline ostium on exam and can have additional cutaneous stigmata, which include hairy nevi, hyperpigmentation, or capillary hemangiomas. If a dermal sinus connects to the subarachnoid space, leakage of CSF will result [2]. Furthermore, in 10-20% of cases, the tract may terminate blindly within the extradural space [13,15].

**Figure 9:**
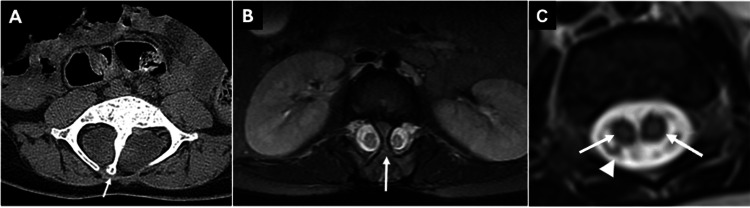
(A) Axial CT image through the lower spine displays findings of a type I diastematomyelia with an osseous septum dividing the spinal canal (arrow) and two separated hemicords. (B) Axial T2 fat-saturated MR image at the level of the kidneys in type I diastematomyelia demonstrating two hemicords separated by an osseous septum (arrow). (C) Axial T2-weighted MR image of the spinal canal shows a single dural sac (arrowhead) containing both hemicords (arrows) in type II diastematomyelia. All images were obtained by the authors from the institutional picture archiving and communication system (PACS). They have been de-identified and used in compliance with institutional policies.

Diastematomyelia: Diastematomyelia is characterized by the formation of two hemicords, which can be either fully or partially divided. It is further classified into two types: type I, where each hemicord is separated by its own dural sac with an intervening osseous or fibro-osseous septum, and type II, where the hemicords are contained within a single dural sac without the presence of an osseous spur (Figure 9) [16]. It occurs most commonly in the lumbar region (over 80% of cases), while upper thoracic clefts are uncommon and cervical clefts are rare [17]. Associated spinal anomalies are seen in as many as 85% of cases [18]. 

CRS: CRS is a rare congenital disorder characterized by a spectrum of developmental anomalies affecting the caudal vertebral column and spinal cord, ranging from isolated sacral agenesis to complete lumbosacral agenesis with multisystem involvement. The extent of spinal cord involvement may not necessarily align with the extent of vertebral column abnormalities [19].

CRS is frequently associated with additional anomalies, including urogenital tract malformations, cloacal abnormalities, lower limb defects, or features consistent with vertebral anomalies, imperforate anus, cardiac defects, tracheoesophageal fistula, renal abnormalities, and limb anomalies (VACTERL) association. It may also be associated with the OEIS complex, which includes omphalocele, bladder exstrophy, imperforate anus or cloacal malformations, and spinal defects. Therefore, following the diagnosis of CRS, a thorough evaluation for associated fetal anomalies is essential [20,21]. Prenatal screening for congenital anomalies offers the advantage of early detection and facilitates appropriate treatment planning, particularly in high-risk cases. The ossification centers of the sacral spine become visible around 16-17 weeks of gestation, which may limit the detection of distal sacral abnormalities prior to this stage on screening ultrasounds [19].

**Figure 10:**
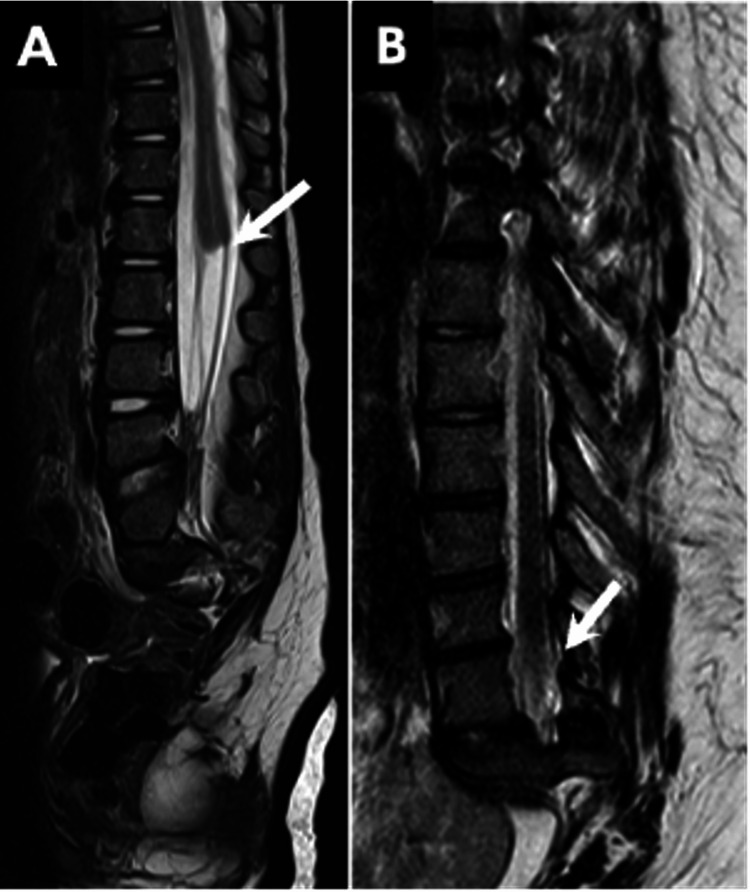
CRS. Sagittal T2W images from two different patients, both with sacral agenesis, illustrate (A) a truncated conus at the T12 level (arrow), characteristic of type I CRS, and (B) an abnormally low conus position (arrow), indicative of type II CRS. CRS, caudal regression syndrome All images were obtained by the authors from the institutional picture archiving and communication system (PACS). They have been de-identified and used in compliance with institutional policies.

CRS can be classified into two types based on the shape and location of the conus medullaris. Type I is characterized by a high and abrupt position of the conus, while type II is defined by a low and tethered conus (Figure 10) [11].

Sacrococcygeal teratoma (SCT)

**Figure 11:**
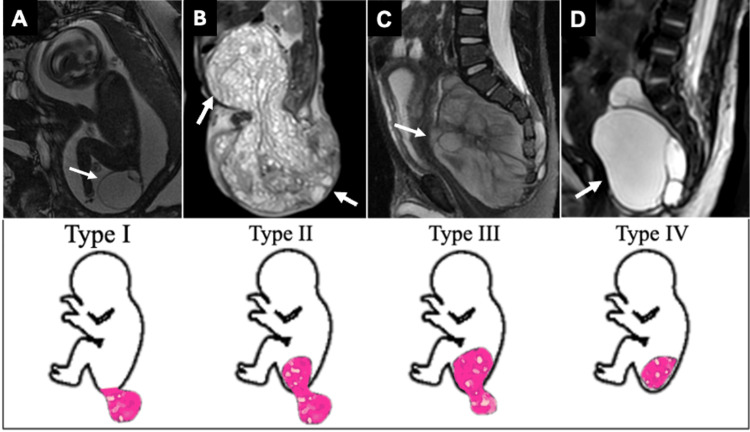
SCT classification according to their location. (A) Type I: Primarily external with a minimal presacral component. (B) Type II: predominantly external but with a notable pelvic extension. (C) Type III: predominantly internal with a small external component. (D) Type IV: Entirely internal with almost no external component. SCT, sacrococcygeal teratoma All images were obtained by the authors from the institutional picture archiving and communication system (PACS). They have been de-identified and used in compliance with institutional policies.

The pluripotent cells of the caudal cell mass can give rise to SCTs, which are the most common congenital tumors in neonates, with a 3:1 predilection for girls [11]. These lesions are extragonadal germ cell tumors, and the majority are benign at birth, with increasing propensity for malignant degeneration with increasing age [22]. They can be seen in isolation or as part of the Currarino triad, comprised of a sacrococcygeal osseous defect, anorectal malformation, and a presacral mass. SCTs have been classified by their location as predominantly external (type I or II) or predominantly internal (type III or IV) tumors (Figure 11).

On imaging, these lesions are classically encapsulated, mixed cystic and solid masses in the lower sacrum, and approximately 5% are predominantly cystic [23,24].

## Conclusions

Congenital spine anomalies include a broad spectrum of developmental disorders, each with distinct clinical implications and prognostic outcomes. These anomalies can have considerable perinatal mortality and long-term neurological consequences in the absence of appropriate recognition and treatment. A solid understanding of their prenatal and postnatal imaging characteristics is critical for timely detection and effective multidisciplinary management. Ongoing advances in imaging technology are further enhancing our ability to identify and characterize these anomalies, ultimately improving patient outcomes. This pictorial review underscores the key imaging characteristics of congenital spinal anomalies. Integrating radiologic findings with clinical context allows for more accurate diagnosis, anticipation of associated abnormalities, and timely initiation of surgical or supportive interventions when indicated.
